# Ecological Facilitation between Two Epiphytes through Drought Mitigation in a Subtropical Rainforest

**DOI:** 10.1371/journal.pone.0064599

**Published:** 2013-05-31

**Authors:** Pei-Yu Jian, Feng Sheng Hu, Chiao Ping Wang, Jyh-min Chiang, Teng-Chiu Lin

**Affiliations:** 1 Department of Life Science, National Taiwan Normal University, Taipei, Taiwan; 2 Department of Plant Biology, University of Illinois, Urbana, Illinois, United States of America; 3 Taiwan Forestry Research Institute, Taipei, Taiwan; 4 Department of Life Science, Tunghai University, Taichung, Taiwan; Centro de Investigación y de Estudios Avanzados, Mexico

## Abstract

Positive species interactions (facilitation) play an important role in shaping the structures and species diversity of ecological communities, particularly under stressful environmental conditions. Epiphytes in rainforests often grow in multiple-species clumps, suggesting interspecies facilitation. However, little is known about the patterns and mechanisms of epiphyte co-occurrence. We assessed the interactions of two widespread epiphyte species, *Asplenium antiquum* and *Haplopteris zosterifolia*, by examining their co-occurrence and size-class association in the field. To elucidate factors controlling their interactions, we conducted reciprocal-removal and greenhouse-drought experiments, and nutrient and isotope analyses. Forty-five percent of *H. zosterifolia* co-occurred with *A. antiquum*, whereas only 17% of *A. antiquum* co-occurred with *H. zosterifolia*. Removing the fronds plus substrate of *A. antiquum* reduced the relative frond length and specific leaf area of *H. zosterifolia*, but removing fronds only had little effect. Removing *H. zosterifolia* had no significant effects on the growth of *A. antiquum. H. zosterifolia* co-occurring and not co-occurring with *A. antiquum* had similar foliar nutrient concentrations and δ^15^N values, suggesting that *A. antiquum* does not affect the nutrient status of *H. zosterifolia*. Reduced growth of *H. zosterifolia* with the removal of *A. antiquum* substrate, together with higher foliar δ^13^C for *H. zosterifolia* growing alone than those co-occurring with *A. antiquum,* suggest that *A. antiquum* enhances water availability to *H. zosterifolia.* This enhancement probably resulted from water storage in the substrate of *A. antiquum*, which could hold water up to 6.2 times its dry weight, and from reduced evapotranspiration due to shading of *A. antiquum* fronds. Greater water loss occurred in the frond-clipped group than the unclipped group between days 3–13 of the drought treatment. Our results imply that drought mitigation by substrate-forming epiphytes is important for maintaining epiphyte diversity in tropic and subtropic regions with episodic water limitations, especially in the context of anthropogenic climate change.

## Introduction

Positive species interactions (i.e. facilitation) play an important role in community dynamics [1], [2] and ecosystem functions [3]. A number of studies have highlighted that interspecies facilitation increases with abiotic factors leading to plant stress [4], [5], [6], [7]. For example, a study of forest restoration in the Mediterranean Basin indicates that nurse shrubs had a stronger facilitative effect on seedling survival and growth on drier than wetter slopes [8]). On a wave-exposed rocky shore, the facilitative effect of goose barnacles on mussel survivorship was positively related to the combination of physical disturbance and thermal stress [9]. However, no studies have examined facilitations among epiphytes or explored the role of such interactions in epiphyte diversity.

Epiphytes contribute up to 30% of species richness in some tropical rainforests and are thus an important component of the extraordinarily high biodiversity in tropical rainforests [10]. Epiphytes lack access to soil water and nutrients released from mineral weathering and litter decomposition. Although N and P have been shown to limit the growth and distribution of plants in forest canopies [11], [12], nutrient limitation on epiphyte growth is not well documented. The lack of direct access to soil water is a key factor confining most epiphytes to humid forest ecosystems. However, even in tropical rainforests, there are rainless periods that may impose drought stress on epiphytes. For example, in a subtropical rainforest in northeastern Taiwan where it rains more than 220 days annually and mean rainfall is 4240 mm annually [13], there were six rainless periods exceeding 7 days over 3 years [14]. Likewise, rainless periods of 8 days or longer occurred 47 times in 2 years in the tropical rainforests of Puerto Rico [14]. Even in forests with high rainfall, many vascular epiphytes exhibit xeric adaptations such as high water use efficiency and low leaf area to dry mass ratios (i.e. specific leaf area, SLA), suggesting that they are often under water stress [15], [16], [17].

**Figure 1:**
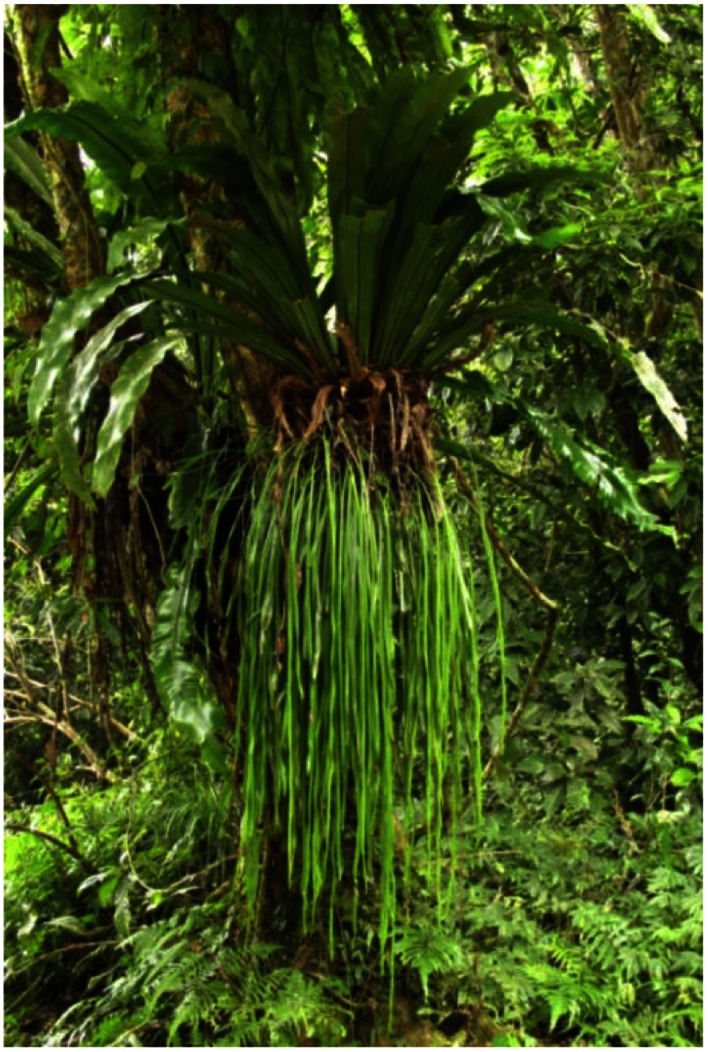
Co-occurring *Asplenium antiquum* and *Haplopteris zosterifolia*. Co-occurring *Asplenium antiquum* and *Haplopteris zosterifolia*.

Humid subtropical forests, which contain abundant epiphytes, cover approximately 58% of the land area of Taiwan. The Fushan Experimental Forest is particularly rich in epiphytes with a total of 65 vascular epiphytes, accounting for 12% of the 515 vascular plants species [18]. Like the rainforests in Costa Rica where as many as 126 epiphyte species have been documented in a single tree canopy [19], epiphytes are often observed in multiple-species clumps in the Fushan Experimental Forest. Many epiphytes may grow together simply because they favor similar shaded microhabitats. However, the frequent co-occurrence of some of the species suggests that other mechanisms may be operating. Dispersal limitations and host-epiphyte interaction have been proposed to explain the clumped distribution or meta-communities of epiphytes [20]. In addition, neighbor’s buffering is probably important in maintaining epiphyte diversity and may explain the clumped distributions of epiphyte species in forest ecosystems [20], [21]. *Asplenium antiquum* Makino (Fig. 1) is one of the most common vascular epiphytes in Taiwan and other parts of the old-world tropical forests [22]. The extraordinary water holding capacity of the substrate formed underneath this species is considered the key factor that allows it to persist in regions with droughts lasting several weeks, such as in the northeastern Australia [23]. The substrate of *A. antiquum* may also benefit co-occurring epiphytes, such as *Haplopteris zosterifolia* (Willd.) E. H. Grane (Fig. 1).

In this study we evaluate the interactions between *A. antiquum* and *H. zosterifolia* in the Fushan Experimental Forest in northeastern Taiwan. We hypothesize that there is a one-way facilitation from *A. antiquum* to *H. zosterifolia* through water and/or nutrient enrichment provided by the substrate of *A. antiquum*. To test this hypothesis, we conducted a field survey on co-occurrence and size association of the two species, and assessed the growth response to reciprocal removal. Furthermore, we tested three potential mechanisms, drought mitigation, nutrient enhancement, and shading, that could have shaped the *A. antiquum* and *H. zosterifolia* interaction through a removal experiment, a drought experiment, and nutrient and stable-isotope analyses. This study offers new insights on the patterns and mechanisms of epiphyte interactions and explores the significance of such interactions in maintaining epiphyte diversity.

## Materials and Methods

### Ethics Statement

Dr. Hsiang-Hua Wang, the director of the Fushan Experimental Forest, issued the permission for conducting the study at the site.

### Study Site and Epiphyte Species

The Fushan Experimental Forest (10 km^2^) is a subtropical rainforest in northeastern Taiwan (24° 34" N, 121° 34′ E). The area has 65 vascular epiphytes belonging to 20 families and 42 genera [24]. Between 1993 and 2007, the annual precipitation of the area varied from 2900 mm to 6650 mm with a mean of 4240 mm [25]. The annual mean temperature was 18.2°C with a low monthly average of 11.8°C in February and a maximum of 24.1°C in July [25]. The relative humidity is above 90% most of the time throughout the year.

Both *A. antiquum* and *H. zosterifolia* are long-lived species abundant in the Fushan Experimental Forest. *A. antiquum* is a litter-basket epiphyte with a bowl-shaped crown that can effectively intercept canopy litter to form humus-rich substrate. The size of *A. antiquum* reaches up to 2 m in crown diameter, but individuals of various sizes are common at our study site [26]. *A. antiquum* exists at all locations on the host trees, from less than 1 m above ground to near the top of the tree canopies and from the trunk to small branches. Like *A. antiquum*, the size of *H. zosterifolia* also varies greatly with frond length reaching up to 1.5 m and the number of fronds as high as >100. In contrast to the bowl-shaped crown of *A. antiquum*, the fronds of *H. zosterifolia* are pendent (Fig. 1).

### Field Survey: Co-occurrence of *A. antiquum* and *H. zosterifolia*

We conducted the field survey on the co-occurrence between *A. antiquum* and *H. zosterifolia* along a first-order stream at Fushan Experimental Forest. We located 100 individuals of *A. antiquum* and 100 individuals of *H. zosterifolia* on the trees starting from approximately 50 m downstream from the weir of Experimental Watershed #1. On the east side of the stream we first identified 50 *A. antiquum* and then 50 *H. zosterifolia*, and on the west side we first identified 50 *H. zosterifolia* and then 50 *A. antiquum*. We surveyed every tree within 1 m off the stream margin. If there was more than one individual of the focal epiphyte species on a tree, we recorded every other one from ground to the canopy top of the host tree. The crown size of each *A. antiquum* was measured, and the 100 individuals were grouped into five categories: <50 cm, 51–100 cm, 101 -150 cm, 151–200 cm, and >200 cm following [26]. The *H. zosterifolia* plants were grouped into four size classes based on the maximum length of their fronds: 40–80 cm, 81–120 cm, 121–160 cm, and >160 cm. We did not include *H. zosterifolia* with a frond length less than 40 cm because they were easily overlooked in high canopies. For each of the 100 individuals of *A. antiquum* and 100 individuals of *H. zosterifolia*, we recorded the host tree species and all co-occurring epiphytes. Epiphytes were considered co-occurring if their roots/stems were in contact with each other.

### Removal Treatment

To examine the interaction between the two epiphytic ferns, we conducted a removal experiment on December 20, 2009 on 55 pairs of co-occurring *A. antiquum* and *H. zosterifolia*. Because of the difficulties in accessing epiphytes high in tree canopies, we only used those that grew less than 5 m from the forest floor. We assigned the 55 pairs into four treatments: *H. zosterifolia* removal (13 pairs), *A. antiquum* frond removal (13 pairs), *A. antiquum* frond plus substrate (frond+substrate) removal (14 pairs), and control (15 pairs in which neither species is removed). *H. zosterifolia* may benefit from co-occurring with *A. antiquum* through enhanced availability of nutrient and water provided by the substrate of *A. antiquum*. In addition, *H. zosterifolia* growing underneath *A. antiquum* may benefit from reduced evapotranspiration due to the shading provided by the fronds of *A. antiquum*. Therefore, we had two different *A. antiquum* removal treatments, a frond removal treatment and a frond+substrate removal treatment. Because the roots of *H. zosterifolia* often grow into the substrate of *A. antiquum*, it was not possible to remove the substrate completely without damaging the *H. zosterifolia* growing underneath. We removed as much of the substrate as possible while trying to avoid physically damaging the roots of *H. zosterifolia*. The treatments were assigned randomly except for eight pairs growing right next to the trails that were open to the public, which were assigned to the control group to minimize interference from tourists. One-way analysis of variance (ANOVA) showed no systematic difference between these eight pairs and the other seven pairs in the control group in all of the pre-treatment measurements taken in this study (frond number and length, SLA, nutrient concentration and light availability). Thus we combined the measurements from the two control sub-groups.

### Growth, SLA and Foliar Nutrient

To assess the growth response to reciprocal removal, prior to and 5 times after the removal treatment (January, February, April, June and August 2010), we measured the length and number of fronds of *A. antiquum* and *H. zosterifolia*. The frond length was determined on three randomly selected un-grazed and mature green fronds from each individual. A frond was considered mature when all sporangia were brown. The SLA of the frond samples collected in January and August was determined by scanning the fronds and calculating the areas with Image J (National Institutes of Health, USA). During each of the five post-treatment measurements, we collected one complete frond of *A. antiquum* and 4–5 fronds of *H. zosterifolia* for nutrient analysis. The frond samples were stored at 4°C without preservatives, cleaned with de-ionized water to remove dust, fungi and other materials, oven-dried at 80°C for 72 hours, and ground. The concentrations of K, Ca, Mg, and P were determined after ashing and dissolving ash in HCl using ICP-OES (Inductively Couple Plasma Optical Emission Spectroscopy, ICP-OES, Jobin Yvon Horiba Group, NY2000, Edison, USA). Total C and N were determined by dry-combustion using an elemental analyzer (Thermo Finnigan NA1500, Bremen, Germany).

### Light Availability above and below *A. antiquum*

To estimate light availability, we took hemispherical photographs for each pair of the two epiphyte species, in October 2009 prior to, and in December 2009, following the removal treatment at the same positions. The two sets of photographs were taken two months apart because hemispherical photographs cannot be taken when it is raining and it rains very often (more than 220 days per year, [25]) at Fushan Experimental Forest. These photographs were taken at one position above the fronds at the center of the substrate, and at three equally spaced positions around the substrate below the *A. antiquum* fronds but above the *H. zosterifolia* fronds. We determined direct site factor (DSF) and indirect site factor (ISF) for each photograph using HemiView 3.43 [27]. The DSF and ISF are the proportion of direct and indirect (diffuse) solar radiation that is transmitted through the plant canopy and reaches the location of measurement [28], [29].

### Water-holding Capacity and Drought Response of *A. antiquum*

To examine the effects of the fronds of *A. antiquum* on reducing substrate water loss through shading, we conducted a drought experiment. Ten individuals of *A. antiquum* with their substrates containing adventitious roots and organic materials were obtained from separate tree trunks at the Fushan Experimental Forest. All plants with their attached substrate were then transplanted to the greenhouse at Tunghai University, Taichung, Taiwan. To ensure similar initial water status of each individual before drought treatment, we saturated the substrates of all plants with water for 3 days.

To test the effect of *A. antiquum* fronds on the water availability of the substrate, the fronds of five of the ten individuals were clipped. The weights of clipped and unclipped epiphyte+substrate replicates were measured daily at 4 pm throughout the drought experiment. The drought experiment was performed inside the greenhouse where relative humidity was maintained above 90% (except on days 17 and 18, with daytime relative humidity of 79% and 88%, respectively) to mimic the environmental condition under the canopy of Fushan Experimental Forest. All plants, with or without fronds, were not watered for 20 days to simulate a drought. Air temperature inside the greenhouse was modulated by an automatic ventilation system; mean daytime and nighttime temperatures were 25.1°C and 23.0°C, respectively. After the drought treatment, the fronds in the unclipped group were clipped. The substrates of both groups were re-saturated with water. After measuring the weight of each substrate under field capacity, all substrates and fronds were dried separately to constant weight.

Because water stored in the substrate of *A. antiquum* probably plays an important role on its interaction with *H. zosterifolia* we measure water-holding capacity of the substrate. The maximum water-holding capacity of each substrate was determined by subtracting the dry weight of the substrate from that of the substrate under field capacity. The fresh weights of the fronds in the unclipped group were determined by subtracting the substrate weight under field capacity from the total weight at day 0 (maximum water holding status of the whole plant).

The potential quantum yield (F_v_/F_m_) was measured daily around 10 am on one randomly selected frond of each frond-unclipped plant using pulse-amplitude modulated photosynthesis yield analyzer (MINI-PAM; Heinz Walz GmbH, Effeltrich, Germany). Leaves were dark-adapted for 40 minutes before measurements. The F_v_/F_m_ was calculated as F_v_/F_m_ = (F_m_−F_0_)/F_m_, where F_0_ is ground fluorescence level of dark adapted leaf and F_m_ is the maximum fluorescence yield of leaf when a saturating light pulse of 10000 µmol m^−1^ s^−1^ is applied for 0.8 second.

### Isotope Analysis

To examine the effects of *A. antiquum* on the water and nitrogen availability of *H. zosterifolia*, we performed isotopic analyses of carbon and nitrogen on (1) the fronds of nine pairs of co-occurring *H. zosterifolia* and *A. antiquum*, (2) the fronds of nine individuals of *H. zosterifolia* without co-occurring *A. antiquum,* and (3) the substrate of each of the nine *A. antiquum*. Two fronds and approximately 100 g of substrate were collected from each sampled individual. Four of the nine samples of each of the three groups were collected on March 2011 and the other five collected on June 2012. These samples were dried at 80°C and ground to powder for elemental and isotopic analyses in the laboratory. About 3 mg of each dried sample was weighed into a tin capsule. Total carbon and nitrogen percentages were measured with a Costech 4010 Elemental Analyzer. Stable isotopes of carbon and nitrogen were determined with a Delta V Advantage, Thermo Fisher isotope ratio mass spectrometer. Isotopic ratios were reported in δ notation (δ = ([R_sample_/R_standard_]–1) • 1000‰, where R = ^13^C/^12^C or ^15^N/^14^N). Analytical precision was better than 0.3‰ for both δ^13^C and δ^15^N on laboratory standards and on duplicate samples. Isotope ratios were expressed relative to the international standards: Vienna Peedee Belemnite (VPDB) for δ^13^C and atmospheric N_2_ (AIR) for δ^15^N.

### Statistical Analysis

We used a Chi-square test to examine if the frequency and size-class distributions of *A. antiquum* and *H. zosterifolia* differed among the three groups (growing alone, co-occurring with each other, and co-occurring with other epiphytes species). We used one-way ANOVA to compare the frond length and number of the two epiphytes before the removal treatment among the treatments. We compared light availability prior to the removal among the four treatments and between above and below *A. antiquum* using two-way ANOVA with treatments and positions as the independent variables. A similar analysis was conducted for light availability after the removal treatment. Because there was a significant treatment x position effect, we compared light availability above and below *A. antiquum* for each treatment using paired *t*-test. We compared changes in light availability below *A. antiquum* following the removal among the four treatments using one-way ANOVA.

We used one-way ANOVA with repeated measurements to compare frond number and length of 1) *A. antiquum* between the control group and *H. zosterifolia* removal group, and 2) *H. zosterifolia* among the control group, the *A. antiquum* frond removal group and *A. antiquum* frond+substrate removal group for the five post-treatment measurements. In the analysis, the frond number and length were expressed as the ratio between each of the five post-removal measurements to the pre-removal measurement. This adjusts for pre-existing differences prior to the treatment although the pre-existing differences were insignificant as will be shown in the result section. We also used one-way ANOVA with repeated measurements to compare nutrient concentration of 1) *A. antiquum* between the control and *H. zosterifolia* removal groups, and 2) *H. zosterifolia* among the three treatments. When among-group differences were significant, we used Fisher’s least significant difference (LSD) tests for post-hoc comparisons. We compared SLA of 1) *A. antiquum* between the control and *H. zosterifolia* removal groups using a *t*-test, and 2) *H. zosterifolia* among the three treatments in January using one-way ANOVA. We used *t*-tests to examine the difference in SLA of *H. zosterifolia* and *A. antiquum* between January and August 2010 for each of the treatment groups. We did not use paired-*t* test because the SLA in the two different months were measured on different fronds.

The daily difference of the relative weight in *A. antiquum* to fully saturated condition between fronds clipped and founds unclipped groups was examined using t-test. We assessed the temporal trend of Fv/Fm using simple linear regression. The differences of both δ^13^C and δ^15^N among four groups (*Asplenium* substrate, *Asplenium* frond, *Haplopteris* growing alone, and *Haplopteris* co-occurring with *Asplenium*) were tested using one-way ANOVA followed by Fisher’s LSD tests for post-hoc comparisons when the among-group differences were significant.

## Results

### Association between *H. zosterifolia* and *A. antiquum*

Of the 100 *A. antiquum* plants, 19 did not co-occur with any other epiphyte species, and the other 81 co-occurred with a total of 18 epiphyte species. *H. zosterifolia* was the second (17 individuals) most common epiphyte co-occurring with *A. antiquum*, following *Pothos chinensis* (Raf.) Merr. (Araceae) (28 plants) and tying with *Procris laevigata* Bl. (Urticaceae) (also 17 plants). Similarly, only 12 of the 100 *H. zosterifolia* plants did not co-occur with other epiphytes, and the other 88 *H. zosterifolia* co-occurred with a total of 18 epiphyte species. *A. antiquum* was the most common epiphyte co-occurring with *H. zosterifolia*. A total of 45 of the 100 *H. zosterifolia* plants existed underneath *A. antiquum*. The frequency distribution of the two species in the three groups: growing alone, co-occurred with each other, and co-occurred with other species, differed significantly (Chi-square = 20.9, *df* = 2, *p*<0.001). The number of *A. antiquum* co-occurring with *H. zosterifolia* was significantly lower than expected from evenly distributed among the three groups, whereas the number of *H. zosterifolia* co-occurring with *A. antiquum* was significantly greater than expected.

**Figure 2:**
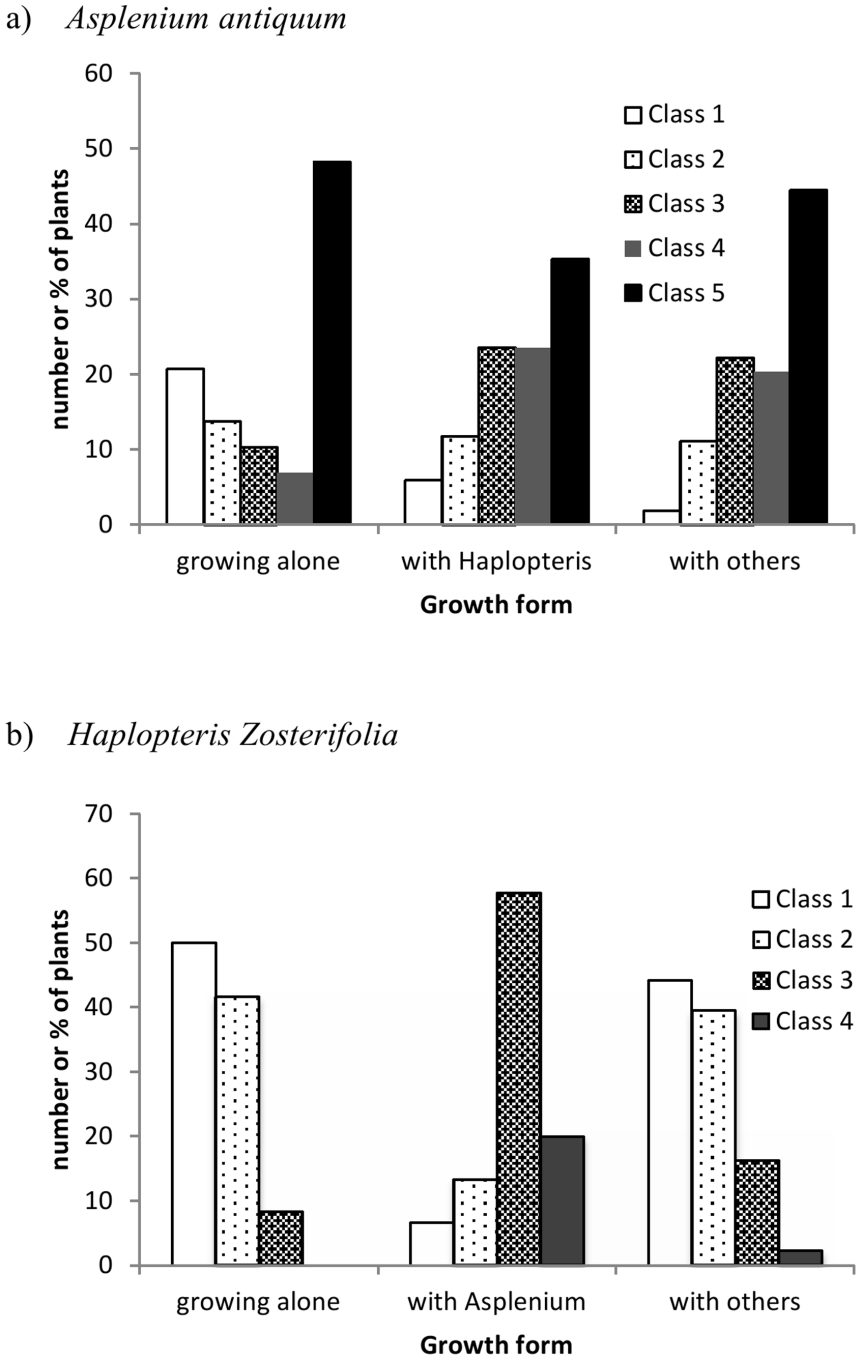
Size class frequency of *Asplenium antiquum* and *Haplopteris zosterifolia* co-occurring with different epiphyte species. Size class frequency of a) 100 *Asplenium antiquum* and b) 100 *Haplopteris zosterifolia* co-occurring with different epiphyte species. Size class of *A. antiquum* was determined by the crown size. 1: <50 cm, 2∶50–100 cm, 3∶101 -150 cm, 4∶151–200 cm, 5>200. Size class of *H. zosterifolia* was determined by the maximum length of fronds. 1∶40–80 cm, 2∶81–120 cm, 3∶121–160 cm, 4: >161 cm.

The size-class distribution did not differ among the three groups for *A. antiquum* (Chi-square = 13.16, *df* = 8, *p* = 0.11; Fig. 2a), but differed significantly for *H. zosterifolia* (Chi-square = 41.9, *df* = 6, *p*<0.001). The *H. zosterifolia* in the group co-occurring with *A. antiquum* had 77% plants in the size classes 3 (120–160 cm) and 4 (>160 cm), whereas those in the other two groups had more than 80% plants in the size classes 1 (40–80 cm) and 2 (80–120 cm) (Fig. 2b). None of the 12 growing-alone *H. zosterifolia* was in the size class 4 (the largest), and only one was in size class 3.

### Light Availability

**Figure 3:**
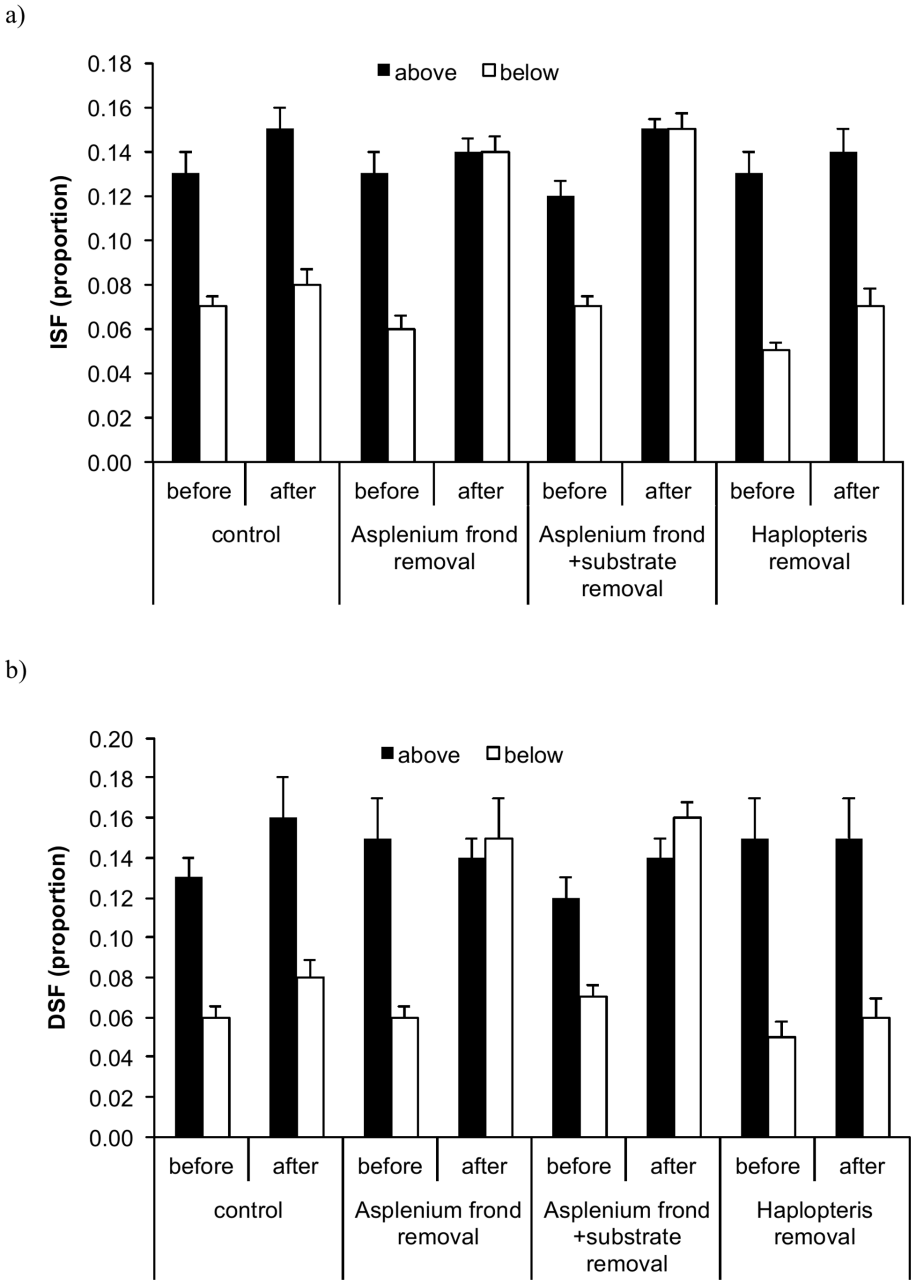
Light indices before and after removal treatment above and below *Asplenium antiquum*. Light indices of a) ISF, and b) DSF before and after removal treatment above and below *Asplenium antiquum*. ISF and DSF are the proportion of direct and indirect light, respectively, at the point of measurement relative to the levels above tree canopies. Error bars represent ±1 S.E.

Prior to the removal treatment, ISF and DSF below *A. antiquum* (0.05–0.07) were approximately 50% lower than the values above it (0.12–0.15) and did not differ among the treatments (treatment effect *F* = 0.49, *df* = 3, *p* = 0.69, position effect *F* = 135, *df* = 1, *p*<0.001 for ISF and treatment effect *F* = 0.32, *df* = 3, *p* = 0.81, position effect *F* = 92, *df* = 1, *p*<0.001 for DSF) (Fig. 3). Following the removal treatment, light availability below *A. antiquum* increased in all treatments possibly due to seasonal changes because the hemispherical photographs were taken two months apart in the winter. However, the increases in both ISF and DSF were more than doubled and significantly higher in the frond removal and frond+substrate groups than the control and *H. zosterifolia* group, which increased less than 40% (LSD tests *p* values <0.001). The post-removal light availability (both ISF and DSF) did not differ between below and above *A. antiquum* in the *A. antiquum* frond removal and frond+substrate removal groups (*p* values >0.05). However, after the treatment light availability was significantly higher above *A. antiquum* than below (*p*<0.001), in both the control and *H. zosterifolia* removal groups (Fig. 3).

### Frond Number and Length

**Figure 4:**
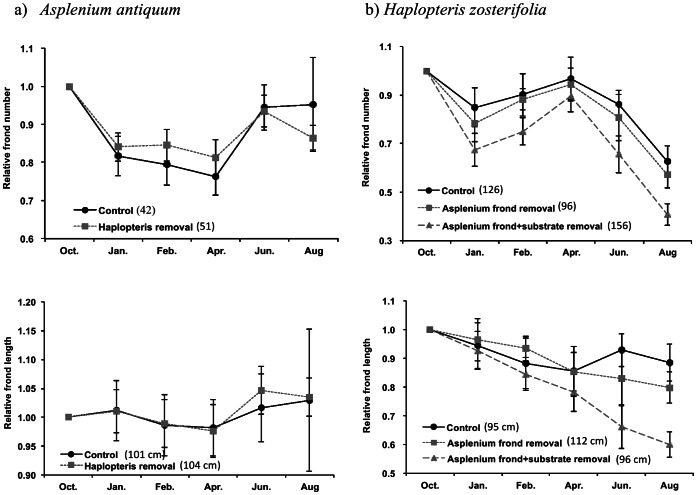
Relative frond number and length after removal treatment. The relative frond number and length (as compared to pre-removal measurements) of a) *A. antiquum* and b) *H. zosterifolia* in different treatments. Error bars represent ±1 S.E. Numbers in parentheses are actual frond number and frond length for October.

Prior to the removal treatment, the frond number and length of *A. antiquum* did not differ between the control and the *H. zosterifolia* removal group (*F*_(1, 26)_ = 2.35, *p* = 0.14 for frond number and *F*_(1, 26)_ = 0.144, *p* = 0.71 for frond length). Similarly, prior to the removal, the frond number and length of *H. zosterifolia* did not differ among the control, *A. antiquum* frond removal group, and the frond+substrate removal group (*F*_(2, 39)_ = 1.11, *p* = 0.34 for frond number and *F*_(2, 39)_ = 2.59, *p* = 0.09 for frond length). Following the treatment, the relative frond number and length of *A. antiquum* (as proportions of the pre-treatment measurements) did not differ between the control group and the *H. zosterifolia* removal group (*F*_(1, 26)_ = 0.008, *p* = 0.93 for frond number and *F*_(1, 26)_ = 0.034, *p* = 0.86 for frond length) (Fig. 4a). Throughout the 10-month period, there was no clear pattern of changes in the frond number and length of *A. antiquum* (Fig. 4a). Thus the presence of *H. zosterifolia* had no effect on the growth of *A. antiquum*.

The changes in the relative frond number of *H. zosterifolia* were consistent and parallel among the three treatments: control, *A. antiquum* frond removal, *A. antiquum* frond+substrate removal. There was an initial decrease between October 2009 and January 2010 and a further decrease between April and August 2010 (Fig. 4b). The relative frond number of *H. zosterifolia* after the treatment did not differ among the three treatments (*F*_(2, 38)_ = 2.11, *p* = 0.13) although the frond+substrate removal group had consistently lower relative frond number. However, the relative frond length of *H. zosterifolia* following the treatments differed significantly among the three treatments (*F*_(2, 39)_ = 5.98, *p* = 0.005). In June and August 2010, frond length was significantly greater in the control group than the frond removal group, which in turn was significantly longer than the frond+substrate removal group (LSD tests *p* values <0.05). Thus *A. antiquum* had a positive effect on the growth of *H. zosterifolia*, and the substrate of *A. antiquum* was an important facilitative factor.

### Elemental Composition

**Figure 5:**
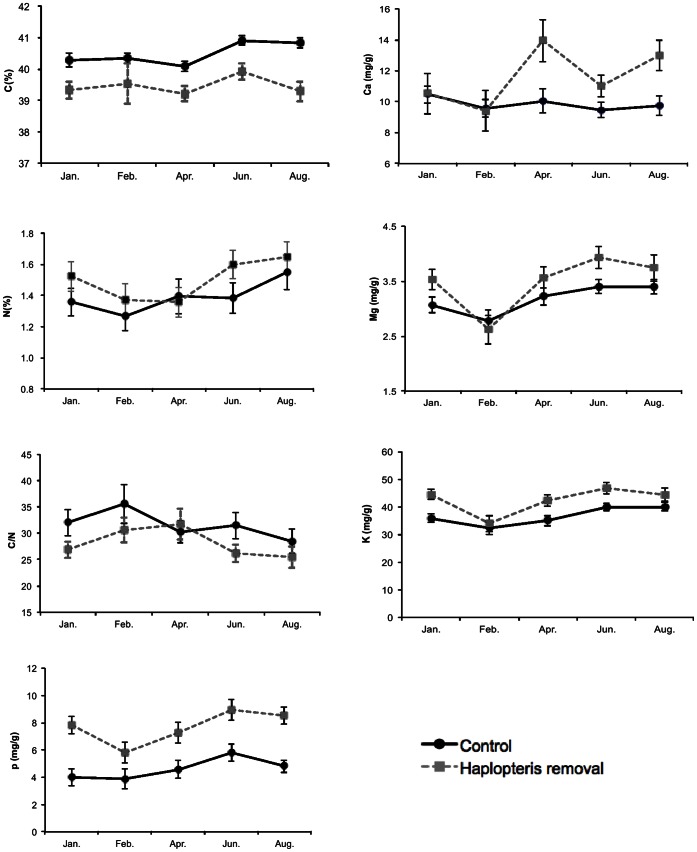
Element content and C/N ratio of fronds of *A. *antiquum. Element content and C/N ratio of fronds of *A. antiquum* between January and August 2010. Error bars represent ±1 S.E.

Concentrations of N and Ca and C/N ratios of *A. antiquum* fronds did not differ significantly between the control group and the *H. zosterifolia* removal group (*p* values >0.05) (Fig. 4). Concentrations of K, Mg, and P were significantly higher in the *H. zosterifolia* removal group than in the control group (*p* values <0.05) (Fig. 5). Carbon was the only element that was significantly lower in the *H. zosterifolia* removal group than the control group (*F*_(1. 21)_ = 13, *p* = 0.002) (Fig. 5).

**Figure 6:**
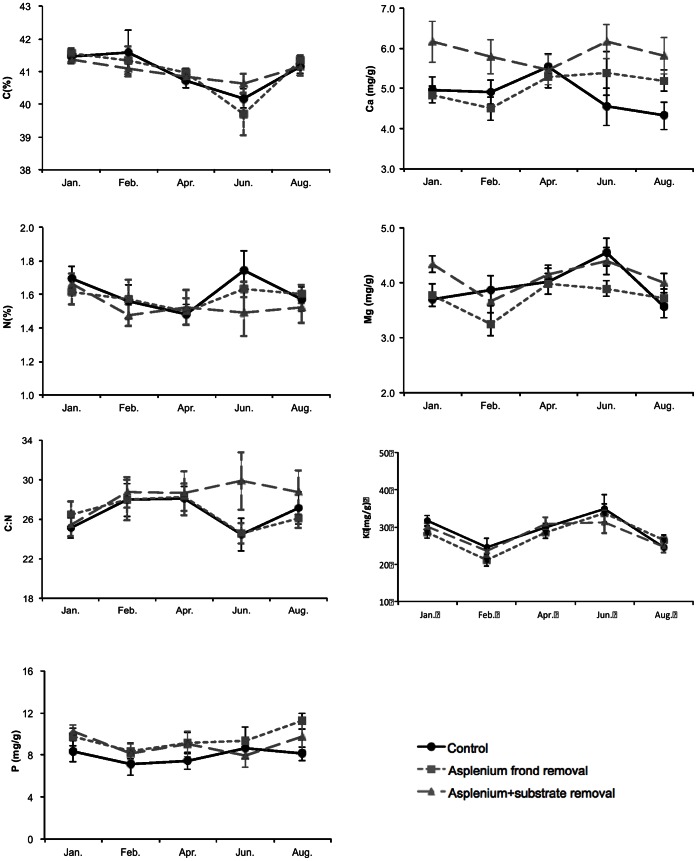
Element content and C/N ratio of fronds of *H. zosterifolia.* Element content and C/N ratio of fronds of *H. zosterifolia* between January and August 2010. Error bars represent ±1 S.E.

For the fronds of *H. zosterifolia*, concentrations of all analyzed nutrients except Ca did not differ among the three groups (*p* values >0.05) (Fig. 6). Ca concentration was significantly higher in the *A. antiquum* frond+substrate removal group than in the control group in January, June and August, and the difference between the control group and the *A. antiquum* frond removal group was only significant in February (LSD tests *p* values <0.05) (Fig. 6).

### Specific Leaf Area

The SLA did not differ significantly among treatments in January 2010 for both *A. antiquum* (*F*_(1, 25)_ = 0.13, *p* = 0.72) and *H. zosterifolia* (*F*_(2, 38)_ *= 0.32*, *p* = 0.73). Between January and August 2010, the SLA of *A. antiquum* increased from 83 to 96 cm^2^ g^−1 ^in the control group and from 87 to 92 cm^2^ g^−1 ^in the *H. zosterifolia* removal group. However, these increases were insignificant both for the control group (*F*_(1, 28)_ = 2.94, *p* = 0.098) and the *H. zosterifolia* removal group (*F*_(1, 22)_ = 0.51, *p* = 0.48). The SLA of *H. zosterifolia* decreased between January and August 2010 in all three groups but the differences were only significant in the frond+substrate removal group (*F*_(1, 24)_ = 17.8, *p*<0.001). In this group, the SLA decreased more than 50% from 230 cm^2^ g^−1 ^in January 2010 to 94 cm^2^ g^−1 ^in August 2010. Thus *A. antiquum* had a positive effect on the SLA of *H. zosterifolia*, but the effect was significant only when both the substrate and fronds of *A. antiquum* were present.

### Drought Stress Response

**Figure 7:**
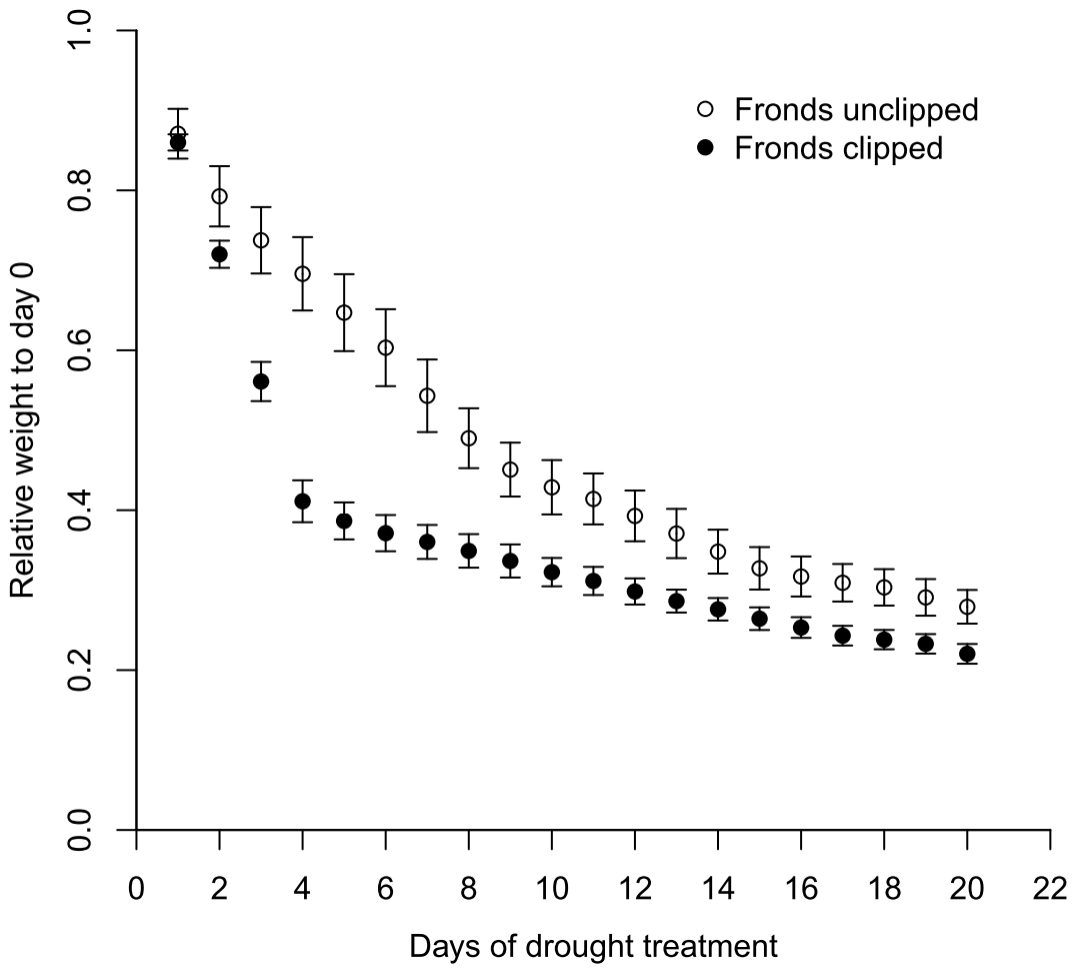
Weight change with the progression of drought treatment. Proportion of weight remaining (relative weight to day 0) with the progression of drought treatment for *Asplenium antiquum* with fronds unclipped (5 replicates) and fronds clipped (5 replicates). The differences between the treatments were mostly significant with the exceptions of days 1, 2, 14, 15, 16, and 19.

**Figure 8:**
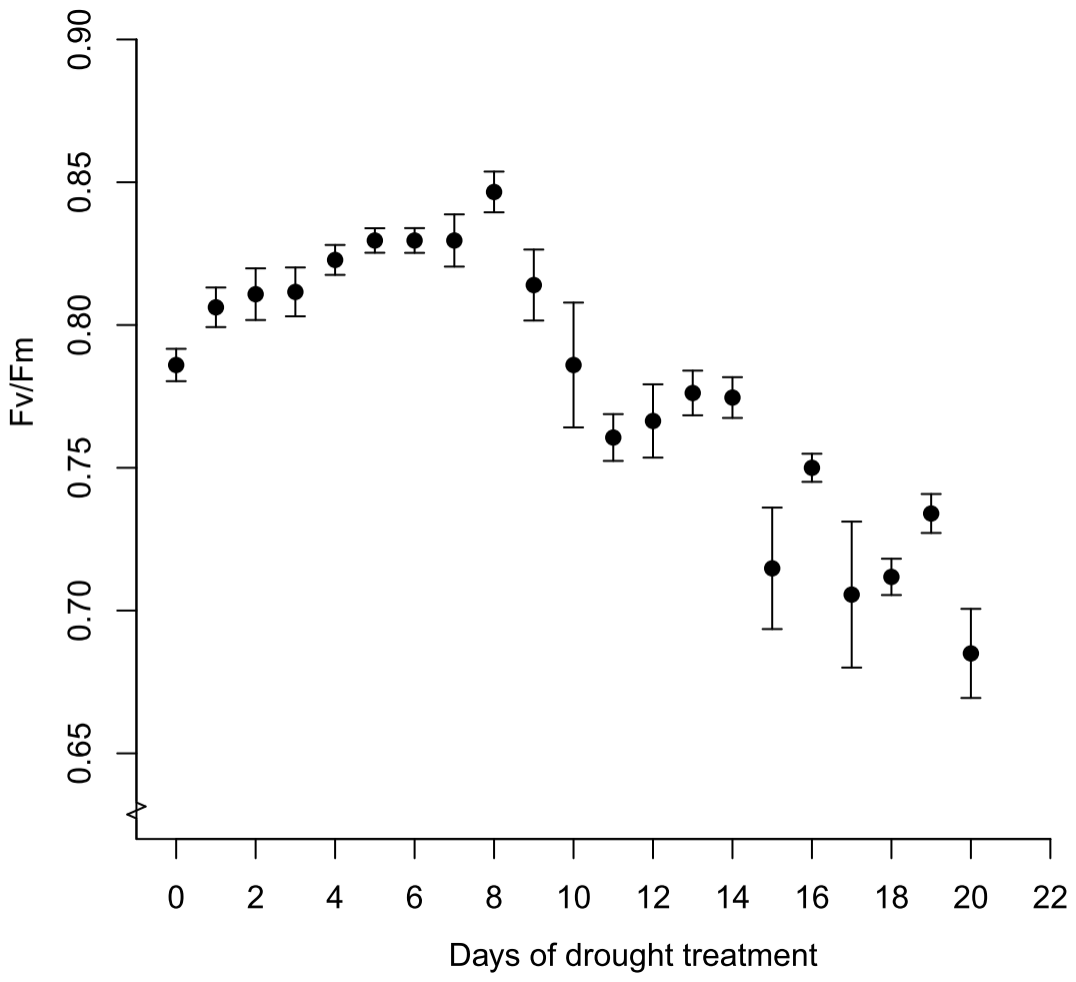
The potential quantum yield of *Asplenium antiquum* with the progression of drought treatment. The potential quantum yield (F_v_/F_m_) of *Asplenium antiquum* fronds with the progression of drought treatment. Error bars represent ±1 S.E.

The maximum amount of water that can be held by the substrate ranged from 4.6 to 7.4 times the dry weight of the substrate, with a mean of 6.2±1.6 (standard error) for the 10 plants. Water loss was significantly greater in the frond-clipped group than in the frond-unclipped group from day 3 to 13 of the drought experiment (*p* values <0.05) (Fig. 7). Thereafter, the difference between the two groups diminished. The F_v_/F_m_ increased gradually from 0.79 to 0.85 before day 8 (*R^2^* = 0.45, *p*<0.0001) and dropped thereafter to 0.69 by day 20 (*R^2^* = 0.58, *p*<0.0001) (Fig. 8).

### Carbon and Nitrogen Isotopes

**Figure 9:**
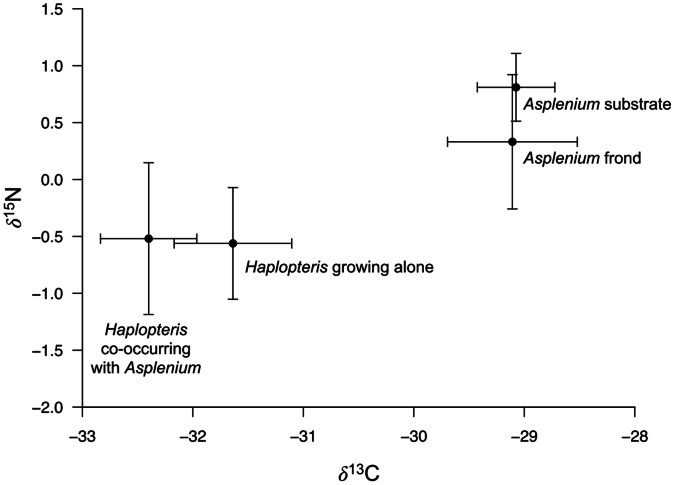
δ^13^C and δ^15^N of *Asplenium antiquum* and *Haplopteris zosterifolia*. Mean δ^13^C and δ^15^N of *Asplenium antiquum* and *Haplopteris zosterifolia*. Error bars represent ±1 S.E.

δ^13^C clearly distinguished *A. antiauum* from *H. zosterifolia* (Fig. 9). The fronds and substrates of *A. antiquum* had similar mean values of δ^13^C (approximately -29‰), which were significantly higher than those of *H. zosterifolia* growing alone (-31.6‰) and co-occurring (-32.4‰) with *A. antiquum* (*p*<0.001). For δ^15^N, no significant differences among groups were found (*p* = 0.21). The two types of *H. zosterifolia*, growing alone and co-occurring with *A. antiquum*, had similar mean values (approximately -0.54‰), which were lower than those of *A. antiquum* fronds (-0.14‰) and substrates (0.12‰) (Fig. 9).

## Discussion

### Effects of *A. antiquum* on *H. zosterifolia*

Forty-five percent of the surveyed *H. zosterifolia* co-occurred with *A. antiquum*, and *A. antiquum* was the epiphyte with which *H. zosterifolia* co-occurred most commonly. The individuals of *H. zosterifolia* co-occurring with *A. antiquum* were significantly larger than those growing alone (Fig. 2), suggesting facilitative effects of *A. antiquum* on the growth and survival of *H. zosterifolia*. The lack of growing-alone *H. zosterifolia* in the largest size class and only one in the second largest size class further suggest that *A. antiquum* and perhaps other epiphytes with which *H. zosterifolia* co-occurred probably also increase the survival of *H. zosterifolia*. This interpretation is supported by the reduced frond length of *H. zosterifolia* resulting from the removal of *A. antiquum*, both frond-only and frond+substrate (Fig. 4b). The reduced frond length of *H. zosterifolia* after the removal of *A. antiquum*, which significantly reduced light available to *H. zosterifolia*, indicates that light was not a main factor limiting the growth of *H. zosterifolia.* The greater decrease of *H. zosterifolia* frond length in the *A. antiquum* frond+substrate removal group than the frond-only removal group (Fig. 4b) implies that substrate plays an important role on the interaction between these two epiphyte species.

*A. antiquum* may benefit co-occurring *H. zosterifolia* through mitigation of water stress. The mitigation may result both from reduced evapotranspiration due to shading of *A. antiquum* fronds (Fig. 3) and from enhanced water availability provided by the substrate of *A. antiquum*. The much higher rates of water loss in the frond-clipped than the frond-unclipped *A. antiquum* in the drought experiment clearly show that shading reduced water loss from the substrate. The removal of *A. antiquum* fronds increases drought stress of *H. zosterifolia* but the effect is even more substantial when the substrate is also removed (Fig. 4). Our SLA data support the interpretation that *A. antiquum* mitigates water stress in co-occurring *H. zosterifolia*. Drought stress and light availability decrease SLA [30], [31]. The more than 50% decrease in the SLA of *H. zosterifolia* in the frond+substrate removal group from January to August 2010 suggests that *A. antiquum* substrates enhance water availability to *H. zosterifolia* where they co-occur.

Foliage δ^13^C is often used as an integrative indicator of water use efficiency, with higher δ^13^C values indicating greater water use efficiency, which is associated with water stress [32]. The slightly greater δ^13^C values of *H. zosterifolia* growing alone than those co-occurring with *A. antiquum* (Fig. 9), though not significant statistically, gives some indication that *A. antiquum* enhances water availability to *H. zosterifolia*. *A. antiquum* is more conservative in water use, as inferred from higher δ^13^C, than *H. zosterifolia*. This conservative water use probably contributes to its widespread distribution even in forests with long dry periods. In contrast, by occurring underneath *A. antiquum*, which shades approximately 50% of the incoming light and provides water through its substrate, *H. zosterifolia* probably lives in a more humid micro-habitat and is thus less conservative in water use.

The substrate of *A. antiquum* may also provide nutrient subsidies to *H. zosterifolia*. For example, epiphytes in lowland tropical forests may be limited by P [12], [33]. However, foliar P concentrations did not differ between *H. zosterifolia* that co-occurred with *A. antiquum* and those that grew alone. Likewise, the N concentrations and δ^15^N values were not significantly different between the two groups (Figs 6 and 9), suggesting common N sources for the two groups of *H. zosterifolia*. Thus our results of elemental and isotope analyses showed no evidence of nutrient enhancement from *A. antiquum* to *H. zosterifolia*.

### Effects of *H. zosterifolia* on *A. antiquum*

Our field survey shows that the distribution of *A. antiquum* was not closely associated with *H. zosterifolia*, as only 17% of the *A. antiquum* co-occurred with *H. zosterifolia* and *H. zosterifolia* was not the epiphyte species that co-occurred most commonly with *A. antiquum*. The lack of size difference among *A. antiquum* that co-occurred with *H. zosterifolia* or other epiphytes, and that did not co-occur with any epiphytes suggests that the co-occurrence of *A. antiquum* with other epiphytes had no effect on the growth of *A. antiquum* (Fig. 2). The lack of significant difference in the frond number and length of *A. antiquum* between the control group and *H. zosterifolia* removal group further supports that *H. zosterifolia* had no effect on the growth of *A. antiquum* (Fig. 4a).

One potential negative effect of co-occurring epiphytes on *A. antiquum* is the competition for water. Our SLA data argue against this possibility, as the SLA of *A. antiquum* increased, although insignificantly, in both the control group and the *H. zosterifolia* removal group between January and August 2010. Thus the water held by the substrate of *A. antiquum* may be sufficient to meet the water demands of itself and its co-occurring epiphytes. However, our drought treatment indicates that if a rainless period persists for more than a week, *A. antiquum* would experience drought stress as evidenced by decreased Fv/Fm (Fig. 8). This stress may occur sooner if the co-occurring *H. zosterifolia* directly competes for substrate water via root uptake. However, *H. zosterifolia* probably has a greater reliance on substrate water than *A. antiquum* due to their differences in water retaining ability associated with different frond morphology. The pendent fronds of *H. zosterifolia* direct intercepted water out of the plant more easily whereas the bowl-shaped crown of *A. antiquum* holds water for a longer period of time. Although the water intercepted by the crown will be directed to the substrate during heavy rainfall events, the water may only be sufficient to moisten *A. antiquum* during light rainfall events in drier periods.

*H. zosterifolia* might have altered the nutrient balance of *A. antiquum*. The higher concentrations of K, Mg and P of *A. antiquum* foliage in the *H. zosterifolia* removal group (Fig. 5) than in the control group imply competition for nutrients in the substrate (Fig. 5). The lower tissue quality of *A. antiquum* in the control group was also indicated by its higher carbon content and low C:N ratio (Fig. 5). Several studies [34], [35] suggest that P is more limiting than N for epiphytes in humid tropical forests, which may explain the consistently greater concentrations of P, but not N, in our *H. zosterifolia* removal group than the control group. However, these nutrient differences did not affect the growth of *A. antiquum* during our 10-month study period. Because the *A. antiquum* could live for more than a decade, our study may not be long enough to detect the negative effect of *H. zosterifolia* on *A. antiquum* resulting from the competition for nutrients in the substrate.

### Positive Interactions between Epiphytic Species, and Implications for Drought Mitigation

Epiphytes are most common in tropical and subtropical regions where rainfall is abundant. However, adaptations such as sunken stomata, succulent leaves, water storage organ, and CAM photosynthesis are common in epiphytes in wet tropical or subtropical forests with high amounts of precipitation [14], [15], [26], [34], indicating that water conservation is important for epiphytes [14], [15], [17,]. Our result suggests that positive interspecific interaction through drought mitigation is another important mechanism that helps epiphytes to survive and grow in the host plants. This positive interaction may also explain the often clumped distribution of epiphytes in the forest canopy.

Crown humus accumulated by epiphytes has been proposed to be an important source of moisture for canopy organisms during dry period [36]. A study of epiphyte biomass at our study site indicates that the humus-rich substrate of *Asplenium* contributed approximately 290 kg ha^−1^ or 8.5% of the total epiphyte biomass [37]. Given that the substrate of *Asplenium* on average holds water approximately 6 times its dry weight, the *Asplenium* substrate has the potential to store as much as 1700 kg ha^−1^ of water in the canopy at scattered spots. Thus substrate-forming epiphytes may play a disproportionally important role in maintaining biodiversity of canopy community by providing a moisture source to facilitate the growth of other epiphytes. In addition to *H. zosterifolia,* the 18 other epiphyte species (22%) co-occurring with *A. antiquum* in our studied forest may also benefit from drought mitigation by *A.antiquum*. Thus substrate-forming epiphytes may function as keystone species, and the microhabitats where they grow are biodiversity hotspots within the forest canopy. A study of forest canopy biomass indicates that five individuals of *A. nidus* hosted a total of 205,428 invertebrates from 27 orders, and the biomass of invertebrates on individual *A. nidus* plants was positively correlated with their number of fronds [38]. At our study site, the biomass of invertebrates was positively correlated with the biomass of *A. nidus* [39]. In Taiwan at least one predatory bird species, *Ictinaetus malayensis,* is known to nest on *Asplenium*. Thus, through habitat cascades [40] they may facilitate the establishment of a highly diverse canopy community.

Interspecies facilitation through drought mitigation may be vital for maintaining epiphyte diversity in the context of anthropogenic climate change. The frequency and severity of drought stress are anticipated to increase in tropical moist forests [41], [42], and as a result drought poses a major threat to biodiversity [43]. Epiphytes have been suggested as early indicators of floristic response to climate change [44] as their abundance and diversity decrease sharply toward drier environments [45]. Ecosystems with exceptionally high epiphyte richness are thus vulnerable to the increasing trend of drought stress [46], [47]. Our study demonstrates the facilitative effect of *A. antiquum* through drought mitigation on *H. zosterifolia*. Such positive interaction between epiphyte species may play an important role in maintaining species diversity in tropical and subtropical ecosystems and in determining their responses to climate change.
